# Organizational Readiness for Implementing an Internet-Based Cognitive Behavioral Therapy Intervention for Depression Across Community Mental Health Services in Albania and Kosovo: Directed Qualitative Content Analysis

**DOI:** 10.2196/29280

**Published:** 2021-11-01

**Authors:** Asmae Doukani, Arlinda Cerga Pashoja, Naim Fanaj, Gentiana Qirjako, Andia Meksi, Sevim Mustafa, Christiaan Vis, Juliane Hug

**Affiliations:** 1 Department of Population Health Faculty of Epidemiology and Population Health London School of Hygiene & Tropical Medicine London United Kingdom; 2 Global Public Health United Kingdom Health Security Agency London United Kingdom; 3 Mental Health Centre Prizren Kosovo; 4 Community Centre for Health and Wellbeing Tirana Albania; 5 Department of Promotion Institute of Public Health Tirana Albania; 6 Department of Public Health Faculty of Medicine University of Medicine Tirana Albania; 7 Department of Clinical, Neuro & Developmental Psychology Faculty of Behavioural and Movement Sciences VU Amsterdam Amsterdam Netherlands; 8 Mental Health Amsterdam Public Health Research Institute Amsterdam Netherlands; 9 World Health Organization Collaborating Centre for Research and Dissemination of Psychological Interventions Geneva Switzerland; 10 European Alliance Against Depression Leipzig Germany

**Keywords:** e-mental health, digital mental health, internet-based cognitive behavioral therapy, implementation science, organizational readiness for implementing change, community mental health center, qualitative interviews, content analysis, Albania and Kosovo

## Abstract

**Background:**

The use of digital mental health programs such as internet-based cognitive behavioral therapy (iCBT) holds promise in increasing the quality and access of mental health services. However very little research has been conducted in understanding the feasibility of implementing iCBT in Eastern Europe.

**Objective:**

The aim of this study was to qualitatively assess organizational readiness for implementing iCBT for depression within community mental health centers (CMHCs) across Albania and Kosovo.

**Methods:**

We used qualitative semistructured focus group discussions that were guided by Bryan Weiner’s model of organizational readiness for implementing change. The questions broadly explored shared determination to implement change (change commitment) and shared belief in their collective capability to do so (change efficacy). Data were collected between November and December 2017. A range of health care professionals working in and in association with CMHCs were recruited from 3 CMHCs in Albania and 4 CMHCs in Kosovo, which were participating in a large multinational trial on the implementation of iCBT across 9 countries (Horizon 2020 ImpleMentAll project). Data were analyzed using a directed approach to qualitative content analysis, which used a combination of both inductive and deductive approaches.

**Results:**

Six focus group discussions involving 69 mental health care professionals were conducted. Participants from Kosovo (36/69, 52%) and Albania (33/69, 48%) were mostly females (48/69, 70%) and nurses (26/69, 38%), with an average age of 41.3 years. A directed qualitative content analysis revealed several barriers and facilitators potentially affecting the implementation of digital CBT interventions for depression in community mental health settings. While commitment for change was high, change efficacy was limited owing to a range of situational factors. Barriers impacting “change efficacy” included lack of clinical fit for iCBT, high stigma affecting help-seeking behaviors, lack of human resources, poor technological infrastructure, and high caseload. Facilitators included having a high interest and capability in receiving training for iCBT. For “change commitment,” participants largely expressed welcoming innovation and that iCBT could increase access to treatments for geographically isolated people and reduce the stigma associated with mental health care.

**Conclusions:**

In summary, participants perceived iCBT positively in relation to promoting innovation in mental health care, increasing access to services, and reducing stigma. However, a range of barriers was also highlighted in relation to accessing the target treatment population, a culture of mental health stigma, underdeveloped information and communications technology infrastructure, and limited appropriately trained health care workforce, which reduce organizational readiness for implementing iCBT for depression. Such barriers may be addressed through (1) a public-facing campaign that addresses mental health stigma, (2) service-level adjustments that permit staff with the time, resources, and clinical supervision to deliver iCBT, and (3) establishment of a suitable clinical training curriculum for health care professionals.

**Trial Registration:**

ClinicalTrials.gov NCT03652883; https://clinicaltrials.gov/ct2/show/NCT03652883

## Introduction

Albania and Kosovo are upper-middle-income countries in the Southeast of Europe. Situated in close proximity to high-income countries with well-resourced health care systems, the state of mental health care in Eastern Europe has gone unnoticed and has been referred to as a global blind spot [1]. In 2017, the burden of mental illness in Albania was estimated to be 3888 per 100,000 people, with disproportionately lower human resource availability, including only 1 psychiatrist, 1 psychologist, and 7 nurses per 100,000 people [2]. In Kosovo, the rate of mental illness has been notably higher due to the conflict that took place in 1998-1999 [3,4]. A survey conducted by the Kosovo Rehabilitation Centre for Torture Victims found that 27.7% of the population reported substantial psychiatric morbidity, in which 22% presented with symptoms of posttraumatic stress disorder, 41.8% for depression, and 43.1% for emotional distress [3]. Another study found that 64.9% of the Kosovan population reported having traumatic experiences during the war, resulting in 200,000-400,000 traumatized persons, which was additional to the existing figure of persons with mental illness [4]. The prevalence of mental illness in postwar Kosovo continues to be a problem, with only a slow decline to show years on after the conflict [5-7]. Despite the high burden of mental illness, the mental health workforce in Kosovo is comparatively lower than that in the neighboring countries, while the country’s mental health budget only equates to 2% of the average mental health budgets of countries in the European region [2].

Limited resources for mental health is, however, not the only barrier toward addressing the care gap. A review of mental health care systems in eastern Europe found that Central and Eastern Europe experienced higher reports of public stigma associated with mental illness compared to other European countries [8]. The high level of public stigma (unwillingness to accept people with severe mental illnesses) may therefore have far reaching implications that can negatively influence policy, funding, recovery, help-seeking behaviors, service quality, and quality of life for people with mental health conditions [8]. A study on the factors that influence access to mental health services in Southeastern Europe (Romania, Bulgaria, and Albania) found that mental health stigma and a lack of knowledge around mental illness were among the factors that influenced delayed access to mental health services [9].

As a response to mental health care access barriers, the use of digital technologies has been put forward as a viable approach for closing the care gap in mental health care owing to the greater potential of technology to expand the mental health workforce, increase access to evidence-based interventions at a lower cost, reduce stigma due to opportunities to engage in treatment remotely, and enable interventions to be linguistically and culturally adapted [10]. Internet penetration rates for Albania (72%) and Kosovo (89%) [11,12] support the argument for harnessing digital technologies in mental health care, as rates appear to be close to or exceed the average internet penetration in high-income countries (75%). However, little to nothing is known about the readiness of mental health services in implementing digital mental health care interventions. Readiness or organizational readiness can be defined as “the extent to which organizational members are psychologically and behaviorally prepared to implement organizational change” [13]. The concept of organizational readiness is an integral component of implementing new health programs owing to the growing recognition that programs may fail not as a result of the digital health innovation but because organizational readiness for change is not adequately evaluated and addressed [14,15]. The assessment of organizational readiness is therefore advised in the early phases of implementation in order to gain a better understanding of the challenges and facilitators for successful implementation, augmentation, and optimization of implementation strategies [16].

The aim of this study was to undertake a qualitative examination of organizational readiness for implementing an internet-based cognitive behavioral therapy (iCBT) intervention for people with depression in 7 community mental health centers (CMHCs) across Albania and Kosovo who were partaking in a large multinational trial on the implementation of iCBT (ImpleMentAll trial, further information about the trial can be found in the Methods section) [17]. To our knowledge, this study will be the first to explore organizational readiness for implementing iCBT as well as a mental health service more broadly in Albania and Kosovo.

## Methods

### Participants and Procedure

Qualitative focus group discussions (FGDs) were conducted with mental health care stakeholders involved on the ImpleMentAll study, a multinational trial funded by the European Union’s Horizon 2020 program. The project aimed to (1) develop and apply tailored implementation strategies for implementing evidence-based iCBT services for common mental disorders in routine mental health care through and (2) conduct a stepped wedge-cluster randomized trial to investigate the effectiveness of tailored implementation, when compared to implementation as usual (more information about the trial can be found in the trial protocol by Bührmann and colleagues [17]). Participants were recruited from 7 CMHCs across Albania (located in Tirana, Shkoder, and Korce) and Kosovo (Prizren, Gjilan, Prishtine, and Mitrovice). Focus groups were conducted prior to the commencement of the ImpleMentAll trial. A purposive sampling method was used for data collection to facilitate access to key informants (mental health care professionals working in and in association with CMHC) and maximum variations within an organization [18,19]) considering diversity across age, gender, job role, and the level of experience of working with people with depression [19]. FGDs were conducted in a meeting room within respective CMHCs.

FGD topic guides were loosely directed by Weiner’s [13] concept of organizational readiness for implementing change (ORIC) [20]. For example, we asked “What kind of training do staff that provide treatment for depression have?” which aims to understand specific organizational resources that are important for the effective implementation of iCBT in services [13]. FGD interviews were facilitated by ACP, GQ, and AM in Albania and ACP, NF, and SM in Kosovo. In Albania, interviews were audio recorded with a digital voice recorder. In Kosovo, the participants requested for discussion not to be audio recorded; therefore, interviews were captured in writing and special efforts were made to document the interviews verbatim in real time. Audio recordings of the interviews were then transcribed verbatim in Albanian and subsequently translated into English. The notes made in Kosovan were also translated to English. This study was approved by the Republic of Albania Ministry of Health Social Protection Ethics Committee on November 17, 2017, and the Republic of Kosovo Qeveria-Vlada Government on February 11, 2017. This project was funded by the European Union’s Horizon 2020 research and innovation program under grant agreement 733025 and received funding from the National Health and Medical Research Council European Union program by the Australian Government (1142363). Funding bodies had no influence on the design of this study. The trial registration number is ClinicalTrials.gov NCT03652883.

### Analytical Framework

**Figure 1:**
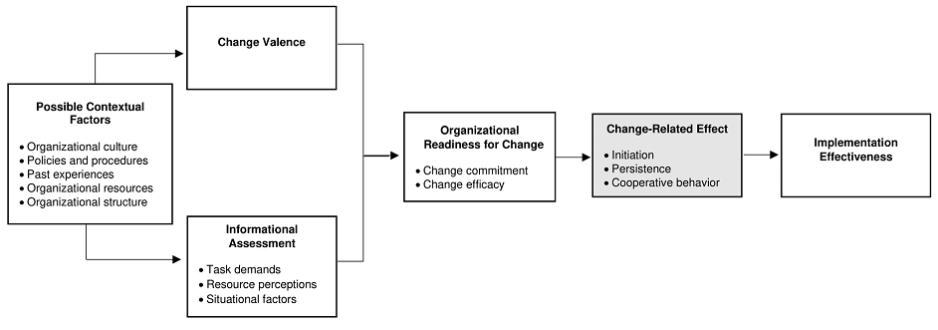
Analytical framework on the determinants and outcomes of organizational readiness for change developed by Bryan Weiner [13].

ORIC, as theorized by Bryan Weiner [13], was used as an analytical framework, as outlined in Figure 1. ORIC [13,21] is a multilevel and multifaceted construct that refers to the organizational members’ shared determination to implement a change (change commitment) and shared belief in their collective capability to do so (change efficacy). Readiness can be theorized, assessed, and studied at the unit of the individual, group, department, and at the organization (eg, CMHC) level. ORIC varies as a function of how much organizational members value the change and how favorably they appraise 3 key determinants of implementation capability: (1) task demands, (2) resource availability, and (3) situational factors. High levels of organizational readiness for change is indicated by organizational members who are more likely to initiate change, exert greater efforts and persistence, and display more cooperative behaviors. The categories outlined in Weiner’s [13] theory were reviewed by the research team to ensure that they align with the preimplementation context that the FGDs took place in. A decision was made to exclude all categories under the change-related effect (grey box in Figure 1), as efforts to initiate change were not experienced at the time in which the FGDs were conducted. Organizational readiness can be assessed across different units, including the individual (individual-level) or the team, department, or organization (supraindividual level). Considering that improvements in health care delivery often involve changes in collective behavior (eg, in staffing, working processes and procedures, decision making, communication), our study will primarily focus on assessing organizational readiness for change at a supraindividual level in relation to the organization across multiple CMHCs that while serving similar clinical populations also vary in context. However, given that opinions may vary between organizations and individuals, our study will also assess organizational readiness for change at an individual level [13,21,22].

### Data Analysis

Data were analyzed at 2 levels: (1) manifest content analysis, which refers to the use of only transcribed interview text, and (2) latent content analysis, which relies on the reflections and interpretations [23]. Thomas and Magilvy [24] suggest that using both types of analyses is important for developing a deep understanding of the data. The unit of analysis for assessing ORIC within the qualitative interview was the CMHC [25]. Prior to data analysis, qualitative data analysts were provided with a sufficient scientific and content-based mastery of the ORIC theory [13], which was used to heuristically guide the data analysis. Definitions of ORIC categories are outlined in Figure 1. The directed content analysis commenced with immersion in the data by all authors of this paper. In order to enhance reliability, the analysis and coding were carried out independently and simultaneously in Albanian (ACP, GQ, AM, NF, and SM) and in English by AD as well as researchers from the ImpleMentAll trial who were not directly involved in the study (CV and JH) [26]. The involvement of independent researchers attempted to include and integrate varying perspectives in the process of data analysis. Transcripts were analyzed in the original language by Albanian-speaking researchers to enable cultural interpretations that may be diluted during translation. This involved reading and reviewing the transcripts several times while considering the following questions: “Who is telling?” “Where is this happening?” “When did it happen?” “What is happening?” and “Why?” [18]. These questions enabled the analyst to steep the data in meaning [18]. Considering different morphological characteristics of the Albanian and English language, line-by-line analysis was impossible and noncomparable between languages; therefore, sentence-by-sentence initial descriptive open coding was carried out for both Albanian and English transcripts. Once the analysts were fully immersed in the data, coding began by identifying and categorizing all instances of ORIC categories. Operational definitions of ORIC categories were developed to ensure systematic analytical processes, given that more than one researcher was involved in the analysis. The transcripts were read through and all texts that on first impression appeared to represent an ORIC category were highlighted. Highlighted passages were then coded using the predetermined codes. Any text that could not be categorized with the initial coding scheme was given a new code. Following this phase, the research team reviewed the data for each category to determine whether subcategories were required. Data that could not be coded into an ORIC theory category but that were relevant to organizational readiness was reexamined to describe different types of organizational readiness. Key emerging themes were mapped into a framework that was reviewed and confirmed by all authors [20,23].

## Results

### Participant Characteristics

Six FGDs were conducted between November and December 2017 in Albania (n=3) and Kosovo (n=3). FGDs included 9-12 participants. In total, 69 professionals participated in this study. Participants represented a spectrum of health professionals working at and in association with CMHCs, with nursing profession accounting for the highest number of participants (25/69, 38%), followed by social workers (13/69, 19%), psychiatrists (13/69, 19%), psychologists (11/69, 16%), general practitioners (GPs) (4/69, 6%), occupational therapist (1/69, 2%), and speech and language therapist (1/69, 2%). GPs external to CMHCs were invited to the FGDs to explore referral routes to CMHCs. Across all sites, 70% (48/69) of the participants were females, and the average age was 41.3 years (range 25-64 years). Managerial positions were held by around 20% of the participants (14/69). On average, years of work experience in mental health was 8 years in Albania and >15 years in Kosovo (see Table 1, for participant characteristics). On average, each FGD took 60 minutes in Albania and 80 minutes in Kosovo. Ahead of the FGDs, participants were asked to rate the following question on a scale of 1-10: “Do you feel the iCBT service delivery will become a normal part of your work?” (a single question that was integrated into their sociodemographic form). On average, both the Albanian and Kosovan sites rated the implementation of iCBT highly, with average scores of 8.42 and 7.48, respectively, out of a score ranging between 1 and 10. Responses for this question were provided by all participants, with the exception of 4 participants from Prishtine and Mitrovice (n=1) and Gjilan (n=3) FGDs.

**Table 1:** Participant characteristics across Albanian and Kosovan sites.

Characteristics			Albania									Kosovo						
			Tirana		Shkoder		Korce		Total		Prizren			Gjilan		Prishtine and Mitrovice		Total
Participants per site (n)			12		9		12		33		15			10		11		36
**Gender^a^ (n)**																		
	Female	9		7		8		24		9			6		9		24	
	Male	3		2		4		7		6			4		2		12	
Age (years), mean (SD)^a^			34.2 (5.65)		36.1 (8.89)		39.3 (10.03)		35.85 (8.20)		46.47 (8.66)			44.88 (7.85)		46.6 (8.92)		46.12 (8.32)
Years of experience, mean (SD)			7.3 (2.09)		5.9 (3.36)		10.3 (8.49)		8.01 (5.71)		>15^b^			>15^b^		>15^b^		>15^b^
Managerial position (n)			0		1		1		2		5			3		4		12
**Professions (n)**																		
	Psychologists/counsellor	3		2		2		7		1			1		2		4	
	Psychiatrists	0		2		2		4		3			2		4		9	
	Nurses	2		4		6		12		6			5		3		14	
	Social workers/psychosocial counsellor	4		1		2		7		4			1		1		6	
	General practitioner	1		0		0		1		1			1		1		3	
	Occupational therapists	1		0		0		1		0			0		0		0	
	Speech and language therapists	1		0		0		1		N/A			0		0		0	

^a^There were missing data for age (n=2) in Gjilan and gender (n=1) in the Prishtine and Mitrovice focus group discussions.

^b^Years of experience in Kosovo were captured using 5 brackets: 0-2 years, 3-5 years, 6-10 years, 11-15 years, and >15 years.

### Qualitative Framework

**Figure 2:**
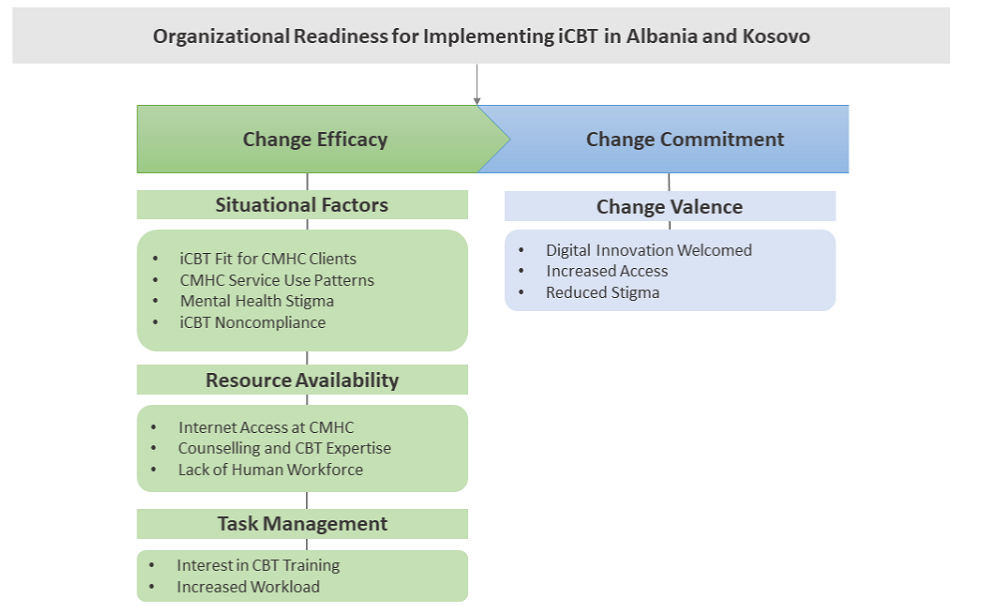
Conceptual framework of the factors that influence organizational readiness for implementing internet-based cognitive behavioral therapy in community mental health teams across Albania and Kosovo. CMHC: community mental health center; iCBT: internet-based cognitive behavioral therapy.

A directed content analysis revealed a multifaceted and multilevel conceptual framework of organizational readiness for implementing iCBT across 7 CMHCs in Albania and Kosovo. Figure 2 outlines the key themes that impacted change efficacy (the belief in the CMHC’s collective/individual capabilities to implement iCBT based on existing situational factors, resource management, and task management of services) and change commitment (individual and shared resolve to implement iCBT based on the perceived value to the service) for implementing iCBT in Albania and Kosovo.

### Change Efficacy Situational Factors

Situational factors refer to CMHCs members’ judgment and perceptions of contextual factors (eg, beliefs, values, habits) that impact the effective implementation of iCBT. The results of the FGDs revealed that the fit and appropriateness of iCBT to clients accessing CMHCs were perceived as both a barrier and facilitator for implementation, while CMHC client service use patterns, mental health stigma, and concerns around iCBT noncompliance were identified as barriers for implementation.

### iCBT Fit for CMHC Clients

FGD participants in Prishtine, Mitrovice, Prizren, Tirana, and Shkoder reported conflicting information about the level of demand for depression-related interventions. One of the participants from Shkoder said:

…Depression is the most common disorder that we see on [a] daily basis. Psychologists provide counselling mostly for depression and other mild mental illness while the rest of the staff, such as the nurses provide treatment for patients with severe or psychotic mental illness that need emergent assistance. Based [on] our statistics, depression has the highest rate in relation to the total number of people who request treatment in our center.

A016

A mental health professional from the same team elaborated that severe mental illness cases received a disproportionate amount of attention, primarily in response to the pattern of service use in CMHCs that appear to be driven by a desire to access welfare services:

…We don’t treat mild and moderate depression because we are focused on treating patients with severe mental illness, [we] help and support their families and provide social and economic assistance to the patients and their families.

A018

Considering all clinical groups accessing CMHCs, participants in the Tirana, Prizren, Prishtine, and Mitrovice FGDs reported that there were very few people seeking treatment for mild to moderate depression. They also reported that people who present with depression are typically seen by a GP and thus not treated with psychotherapy. The FGDs in Tirana and Gjilan reported that CMHCs were largely accessed by people with severe mental illness. Not having enough people access services for common mental disorders was perceived as a barrier for the effective utilization of iCBT. Although all GPs reported that they saw large numbers of people with mild to moderate depression, “We have (see) mild and moderate depression which we refer for treatment to a psychiatrist, we don’t prescribe medication without consulting a psychiatrist first” (A007), this was not reflected in the clinical groups accessing CMHCs:

…we treat mostly patients with major mental health disorders, including psychosis such as schizophrenia, mood disorders with psychotic episodes. We have a small number of patients with anxiety and depressive disorders and other mental health. We have few patients with mild and moderate depression and anxiety disorders.

A005

### CMHC Service Use Patterns

Closely related to the perceived inadequate fit of iCBT for the clinical groups accessing the CMHCs, participants in the Tirana and Shkoder focus groups suggested that there were 2 key patterns for using the CMHC. First, they reported that people generally attended services when their condition was severe and typically discontinued their use of the CMHC when they got better.

…When their (clients’) mental health condition gets worse, they come to us for treatment and when they get better, they stop the treatment because they think that they don’t need to continue the treatment anymore.

A014

…We also have patients who come only once for evaluation and a month-long treatment and then they stop coming to our center…They continue to self-medicate for years buying medicines at the pharmacies over the counter and then they show up again.

A003

Second, participants in the Tirana and Shkoder FGDs also suggested that typical clients were from lower socioeconomic status with severe mental health conditions who accessed the CMHC with the main purpose of applying and qualifying for disability allowance, as CMHCs hold the authority to assess and certify mental health–related disabilities in Albania. Accessing CMHCs therefore appears to function as a means of accessing welfare services:

…That’s why we don’t have patients with mild or moderate depression only, most of them have chronic or major disorders that come for the application of the reimbursement of the medication they take.

A013

…The focus of our work is disability assessment and certification.... and we normally follow up those who we certify as disabled, visit them at home and make sure they take their medication... hence these are the issues we are focusing on.

A005

Participants stated that all CMHCs were situated in major towns, which makes access to services from rural area populations very challenging and that may consequently affect the timely treatment of people with mental illness in the region:

…The problem is related to the access of our services. Those who live in the city have an advantage over those who live in remote areas. They can easily access the service compared to residents who live in rural areas. When patients from remote areas come to us for treatment they have already developed a chronic mental health condition.

A017

…When they come to us they are in a deteriorated state. Some of them come for treatment after attempting suicide or when they are struggling.

A015

When prompted further to explore how information on clinical presentations was recorded upon service access, there appeared to be some uncertainty regarding the number of clients and their diagnosis at the CMHC, in which participants suggested that while individual client files were kept, no specific service-wide records were being collected, reported, or analyzed, which is itself a barrier to implementing iCBT.

…We do not have a database.

K2104

…We don’t have numbers of patients being treated based on their diagnosis.

A013

### Mental Health Stigma

FGD participants across both Albania and Kosovo unanimously reported that self and public stigma in relation to mental health and seeking treatment in mental health care services was high. They mentioned that clients did not want to be associated with a label that could lead to social exclusion, which may prevent people from seeking treatment for common mental health conditions.

…I have an example, three of our patients live in the same building and fear that their neighbors might know about their mental health problems. And so, when we do our home visits, we have to wait for one of the patients to close their apartment door after the visit in order to knock at the other patient’s door to enter in, in order not to be seen that we have been in their houses despite the fact that they know about each other’s mental health issues, and every time we go to their homes they talk about each other.

A004

Interestingly, as described earlier, the primary service is inundated with individuals with depression as this GP accounts:

…Almost, every day we have similar cases with mild depression but who refuse to go to a psychiatrist. They are afraid and withdrawn because they think that it might be a mental illness problem and get scared of what might happen to them if that would be the case. Meanwhile we give them advise but we would like to have a psychologist to handle this kind of work.

A007

They do not want to be referred to CMHCs because of the stigma associated with mental health as well as the institutions that provide such support:

…I think that because of the stigma, people suffering from depression avoid seeking help in specialized mental health center such as this one…

A001

A participant in Shkoder mentioned that it was difficult for participants to accept their diagnosis, leading them to seek different consultations before accepting their condition.

…Usually, they (patients) seek several consultation because it’s difficult to accept a mental health disorder.

A007

Taking this into consideration, it appears as though stigma may be the driving force behind a lack of access to mental health services by people with mild to moderate depression and service user access patterns across Albania and Kosovo.

### iCBT Nonadherence

Another perceived barrier expressed by participants in the Tirana FGD concerned adherence to iCBT. Participants in the Tirana FGD expressed that some patients with depression were difficult to engage. While some participants did not see obstacles in engaging adolescents and adults to iCBT, they expressed concerns around engaging older adults with depression.

…Those who suffer from depression will be very difficult to engage through the iCBT intervention. As for the other patients with different diagnosis I think that it won’t be a problem.

A005

…I don’t see any obstacles for adolescents, while for adults and elder patients it will be difficult.

A014

Perceived concerns around confidentiality and difficulty of engaging people with depression appeared to make participants question whether clients would adhere to the iCBT intervention. Participants also expressed concerns around the clients’ lack of access to the internet and computers.

…The patients we see are very poor and often they come to us to get the disability benefits because it the only income resource for them. The phone is a more appropriate way of getting in touch with them.

A004

Participants perceived that clients may have concerns around confidentiality of the iCBT platform, fearing that their information will be accessed by others. Such concerns stem from the pervasive stigma of using mental health services in Albania.

…. I think that confidentiality will be an issue. It will be difficult to persuade our patients that every data that will be put on the platform will be confidential, at the beginning we would need to explain this.

A003

### Resource Availability

Organizational resources broadly refer to the CMHC’s existing financial, material, human, and informational resources that are used to make a judgement about implementing and delivering iCBT. The FGDs suggested 2 resource facilitators for implementing iCBT, including appropriate counselling and CBT expertise and internet access, and 1 barrier relating to the lack of available human workforce.

### Internet Access at CMHC

Participants across all Albanian FGDs suggested that their CMHCs lacked technological infrastructure and that an investment was required. However, participants in Kosovo reported having a good technological infrastructure in place.

…We have some problems with the internet provider, there are interruptions from time to time.

A021

Participants suggested that setting up iCBT within the service would be feasible should clients wish to access iCBT through the CMHC.

…Of course. It would be a good idea if the patients receive help from the psychologist while getting access to the iCBT. It’s a good thing to have a computer and internet installed in this room and we could help the patients to fill in the worksheets and the patients can come whenever they want in order to make use of the iCBT platform.

A004

### Counselling and CBT Expertise

All FGDs reported having clinical professionals within the team, primarily psychiatrists and psychologists who could be leveraged to implement iCBT. However, most participants with counselling experience (eg, psychologists, psychiatrists) reported that they did not have the necessary CBT accreditation, with those with knowledge around CBT stating that the training they received was either superficial or did not include a practical component. Moreover, participants suggested that they did not have the general supervision infrastructure to support iCBT.

…Besides to what we have learnt in the university classes we have not been trained in CBT.

A021

…We had some hours in our psychotherapy module (in University) but it was a short introduction to CBT and we had to read a (book) chapter by ourselves but no practice.

A015

Participants stated that CBT training could only be accessed privately, requiring clinicians to pay out of their pockets and that training was difficult to access:

…We were notified not long ago about the starting of a training course on CBT, but it was organized in Tirana and the course was very expensive.

A018

…I try to apply CBT and I face a lot of difficulties... I have had private consultation with the only person who is trained on CBT in the city.

A015

The team in Prizren also reported using validated (albeit not in Albanian) psychometric scales to screen and monitor the progress of clients, while the FGD in Tirana reported that assessments for mental health were performed by a psychiatrist as a screening but not monitoring measure. This was deemed to be a strength that would be of help in monitoring client outcomes in iCBT.

…[We have used] Hopkins Questionnaire for Measurement of Depression and Anxiety, Beck Depression Scale, Mini International Neuropsychiatric Interview.

K2205

### Lack of Human Workforce

Despite the presence of a small number of clinical staff with limited counselling or CBT experience, participants in most CMHCs reported feeling overstretched owing to expectations to treat clients with heterogeneous clinical presentations and across a wide geographic radius. A lack of adequately trained staff appears to present a barrier to the implementation of iCBT.

…[we see] All disorders, psychotic disorders, personality disorders, depression, anxiety, phobia etc...

K2206

…We have a geographically heterogeneous patient population (ranging) from the city to the remote villages.

A004

…1/3 city’s center population. We cover 4 or 5 municipal units and its primary health care centers, also some parts of the city’s suburb areas.

A013

…According to the mental health Law, a CMHC should cover 50-150 thousand inhabitants, but in reality we cover 300 thousand inhabitants…threefold to recommended coverage.

A003

### Task Management

Task management refers to the CMHCs’ knowledge of task demands by iCBT, while cognitively appraising the match between task demands that are available. The FGD findings suggested that while interest in CBT training was perceived as a facilitator for effectively managing iCBT tasks, an increase in workload was perceived as a barrier.

### Interest in CBT Training

FGD participants across Tirana, Prizren, Prishtine, and Mitrovice said that they wished to gain CBT skills. Psychologists, GPs, and psychiatrists expressed that they would like to gain new skills, while social workers and nurses who do not typically deliver CBT expressed an interest in being trained.

…If you involve the social workers and nurses as participants on these trainings, then yes.

A006

…Such training would be welcomed as we have not had such training and it would be very beneficial.

K2301

Higher cadre staff such as GPs and psychiatrists were especially keen that staff of a lower cadre such as nurses be trained in CBT, suggesting that it could further enrich the quality of services provided to all clients.

…We work in different ways the counsellor and the nurse give advice to the patient and helps them but none of them work without the permission of [the] doctor. We should train the nurses like most of countries that are free to take the history on its basis... We have 1200 folders and our work is very hard and we want to give service to patients.

K2104

### Increased Workload

Participants in the Shkoder and Tirana FGDs suggested that despite the perceived benefits of introducing iCBT, they anticipated that the project would take a toll on their workload.

…Besides the working benefits that we gain from this project I will have to work extra hours to what I already do here.

A021

Participants in the Tirana and Shkoder FGDs also suggested that they felt used by previous research projects and enquired if monitory incentives would be available for their additional efforts. They also suggested that clinicians should have the opportunity to decide if they wanted to participate in the trial and implementation in accordance with the additional strains that this may take on their work.

…This will be an extra engagement from my side and I have the right to decide if I want to be involved or not in this project… we understand the aim and structure of the project, we would like to know if there is going to be a monetary incentive/wage for the participants. We have had a lot of people who wanted to implement their projects and we have felt used by them.

A021

### Change Commitment (Change Valence)

Change valence broadly refers to the CMHC member’s perceived value of iCBT. FGD data suggested that participants welcomed the innovation of iCBT treatment and valued the role of iCBT for people who are living geographically isolated lives and for reducing stigma associated with accessing mental health services.

### Digital Innovation Welcomed

Participants across the Tirana, Prizren, Prishtine, and Mitrovice FGDs appeared to express optimism and high hopes for the use of iCBT, which participants perceived as innovative and potentially leading to improvements in the choice and quality of services.

…We would all be interested about CBT and iCBT training in order to have a different approach with clients. The online intervention would be most welcomed and knowing that this will be the first time that is being implemented in Kosovo we are ready to support it.

K2101

### Increased Access

FGD participants from Prishtine, Mitrovice, Tirana, and Prizren said that online interventions could lead to an increase in service access for people who are residing in geographically isolated regions, living isolated lives, and people who are living in poverty. It was also reported that the implementation of iCBT could remove access barriers related to travel, thereby increasing access to the CMHCs.

…It will be as an open door and relief for depressed patients who need additional psychotherapy. [iCBT] saves client time (travel to psychotherapist) because eg. Ifightdepression tool can be used in distance.

K2205

…this online method would be very welcome especially for women who live isolated [lives] for different reasons knowing that technology is used from every person and can be treated online without problems so we as a therapist would be very much pleased and satisfied if we were training in this very important field.

K2101

### Reduced Stigma

While stigma was perceived as a barrier for meaningful exposure to iCBT, FGD participants from Tirana, Prizren, Prishtine, and Mitrovice collectively suggested that the use of iCBT could reduce the stigma associated with mental health care access. For instance, participants reported that clients were concerned about being seen accessing the CMHC, fearing how this might be perceived by their wider community.

…Sometimes, they might come at the treatment center at the same time and pretend to not know each other.

A005

Participants reported that some of the CMHCs also serve as day centers for long-term community clients, reinforcing that centers mainly support people with severe mental health conditions, thereby increasing the stigma experienced by clients. The versatility of a web-based CBT intervention could allow clients to engage in treatment for depression at home where they may feel more comfortable and less stigmatized.

…It (iCBT) would be very successful by knowing that stigma is very present, there is always a problem to convince patients to take the right services and this is the reason why this project will have success. For example, if a patient comes to [name of service], they can see patients with serious mental illness here staying in Day Center which increase fear and stigma and if I will show them this web-based tool I’m very sure they will feel more comfortable working from home.

K2206

## Discussion

### Principal Results

We conducted a qualitative examination of organizational readiness for implementing an iCBT intervention for people with mild to moderate depression with health care professionals from 7 CMHCs across Albania and Kosovo. Our directed qualitative content analysis revealed 2 overarching themes of organizational readiness for implementing iCBT that aligned with Weiner’s [13] ORIC model that we used to interpret the data heuristically. The first was change efficacy, referring to the perception of how possible it would be to implement iCBT in relation to 3 themes, namely, situational factors, resource availability, and task management, which were largely perceived as barriers. The second theme was change commitment, which included 1 subtheme, change valence, in which participants largely expressed that iCBT could result in benefits to their organization and the communities they serve.

### Comparison With Prior Work

Several situational factors were identified as barriers for implementing iCBT for depression. Most participants suggested that iCBT was not a good fit for the clinical populations using the CMHCs, which largely consisted of clients with severe mental illness (eg, schizophrenia, mood disorders with psychotic episodes). Moreover, it was reported that other clients accessing the CMHC discontinued their use of the service once their condition was perceived as manageable. These patterns of service use appear to be consistent with the treatment prevalence for psychoses being higher when compared to the treatment prevalence of depression in upper-middle-income countries in contrast to that in high-income countries in which rates are comparable. This demonstrates that people with depression may be underserved in the region [2]. Participants from the Albanian FGD also reported that people with mental health conditions with a low socioeconomic status were more likely to access services as a way of applying for disability allowance and in which access to the CMHC provided a means of accessing social support. While data from the FGDs appear to indicate that stigma is the driving force for the lack of poor uptake by people with low-to-moderate presentation of mental illness, the lack of specialist community mental health services to address common mental disorders may also deter people from accessing services. This is supported by research that perceiving treatment or services as undesirable can lead to delays in access to treatment [9].

Participants expressed concerns that their clients may fear data privacy breaches and that this may negatively impact access to iCBT. Similarly, a qualitative study exploring the views of people with severe mental illness on digital mental health interventions [27] found that participants expressed concerns about the privacy and confidentiality of their data. Nevertheless, participants also reported that they would still be willing to engage in digital mental health interventions if they felt fully informed and reassured about the use of their data [27]. Increasing transparency around how client data are processed may therefore be helpful in reducing fears around data privacy. While the clinical presentations of people accessing CMHCs appear to be a barrier for implementing and delivering iCBT for depression, some participants (particularly GPs) suggested that they were seeing a high volume of people with symptoms of depression that were hesitant to accept a mental health diagnosis. Participants appeared to link the low uptake of services to the high levels of stigma associated with mental health care in the region. Social stigma of mental illness has been reported globally, not only in Eastern Europe. A review of mental health care systems in Eastern Europe found that experiences of public stigma were higher in countries in Central and Eastern Europe as compared to those in European Union countries [8]. The high social cost of mental illness may therefore negatively impact a person’s access to treatment and their mental health trajectory [8]. Remote access to mental health interventions such as iCBT may therefore be critical in reducing the stigma associated with in-person interactions within brick-and-mortar mental health services. A survey on mental health service utilization in Southeastern Europe that included Albania found that perceived stigma led to delays in accessing services as long as 3 months [9]. Taken together, these findings support reports by participants in our study that service access may be negatively impacted by the pervasive stigma associated with mental illness and treatment-seeking behaviors in the region.

Public stigma appears to negatively impact how people access mental health services, requiring significant shifts in public perception around how mental health and treatment access are perceived [28]. Public-facing campaigns such as television programs that raise awareness of mental health treatment or that raise the voices of those experiencing mental health conditions have been put forward as a possible solution for addressing mental health stigma [9]. While evidences regarding the effectiveness of public-facing antistigma interventions in low- and middle-income countries are limited [29], there is some evidence to suggest that they can be effective. A study evaluating the impact of a brief television news report in reducing public stigma associated with schizophrenia in Brazil found that stigma scores were significantly lower in relation to the desire for social distancing and restrictions to the rights of people with schizophrenia after watching the news report vignette and when they were followed up at 3 months [30]. Promising evidence also comes from an antistigma program (Time to Change Global), which aimed to tackle stigma and discrimination toward people with mental health problems in Ghana and Kenya. The project found significantly positive changes in stigma-related desire for social distancing from people with mental health problems, intent to contact with people with mental health problems, and stigma-related knowledge after a short-term public campaign [31].

Participants reported both facilitators and barriers in relation to resource availability for implementing iCBT. The general technological infrastructure required for iCBT (eg, access to computers, internet at the service) was present in Kosovo but not in Albania where substantial investment was required. Participants said that there were clinical professionals within the team (ie, psychologists and psychiatrists) who either had CBT or counselling skills that would enable them to deliver the intervention. However, when probed further, they explained that their CBT skills were limited and gained by reading book chapters during their university courses, with no clinical practice or supervision. With the exception of 1 psychiatrist in Kosovo who had received formal training, none of the 11 psychologists and 13 psychiatrists had received clinical training on CBT or other psychotherapy approaches, as clinical training and practice are not included in the academic curriculum for Clinical Psychologists in either Albania or Kosovo. Consequently, CBT training was welcomed not only by psychologists and psychiatrists but also by a range of mental health professionals, including those who did not traditionally deliver CBT, such as nurses and social workers. Numerous studies have identified similar barriers in relation to the implementation of iCBT. A qualitative study on the facilitators and barriers of delivering blended CBT (therapist plus computerized program) in the United Kingdom found that the intervention was found to be time consuming, disruptive to usual practice, and overwhelming owing to having low perceived confidence and practice in supporting clients through iCBT [32]. The provision of e-mental health interventions appears to be the simplest part of implementing blended CBT, warranting the development of a toolkit that can enable implementation to be tailored around service needs [33]. Moreover, it is imperative for academic curriculums of Clinical Psychology MSc level courses in Albania and Kosovo to integrate clinical training in their syllabus as well as adopt service-level adjustments that permit staff with the time, resources, and clinical supervision to deliver iCBT in order to increase organizational readiness and facilitate the effective implementation of iCBT in Albania and Kosovo.

Participants also suggested that staff were overstretched by providing services for populations up to threefold more than that recommended by law. Barriers to task management were also associated with a high workload and lack of incentives or compensation to take on additional tasks. The lack of resources and availability of specialist mental health facilities often mean that community mental health services are required to cater for a broader range of people. It has therefore been suggested that transdiagnostic interventions may have wider feasibility and application within low- and middle-income countries [33]. Adopting a versatile iCBT program can be used for a range of different disorders to increase utility in day-to-day practice. Notwithstanding the barriers for implementing iCBT, participants appeared to welcome innovations in the service, perceiving a range of benefits for isolated and stigmatized clients that enabled greater access to mental health services. A metasynthesis review from high-income countries on the user experience of iCBT found that client participants appreciated the privacy and access that it allowed for people who felt stigmatized by mental health services or those who experienced mobility problems due to a physical disability [34].

### Limitations and Strengths

Our study had several limitations. While Albania and Kosovo are located in the same region and have the same language, culture, and values, they are 2 distinct countries with different health care systems that are presented with different challenges and demands. Data for both settings were merged because there were not enough data to conduct a separate analysis for each country. As a result, contextual or nuanced interpretation of the data could not always be generated. We attempted to address this limitation by indicating which sites endorsed different themes. Moreover, the qualitative data analysis did not reveal any conspicuous differences between the sites. Nevertheless, many commonalities were discovered, allowing for greater generalizability to be made. The CMHCs that implemented iCBT mainly provided mental health services for people with severe mental illness because there are little-to-no publicly available psychological services. The mismatch between people who access CMHC services and the target population for iCBT may have negatively impacted perceptions around the feasibility of the implementation of iCBT, even though participants reported that they would value the use of digital innovations in their service. Nonetheless, implementing iCBT within CMHCs was the most feasible option in a region that has limited mental health resources.

This study also had several strengths. Our study is the first to examine organizational readiness for implementing a digital mental health intervention in both Albania and Kosovo. A wide range of professionals participated in the qualitative interviews, representing all professions working at and in association with a CMHC. There was also a good geographical representation of services across both Albania and Kosovo. The findings of our study provide a unique contribution to the literature in relation to the barriers and facilitators for implementing iCBT in these regions and can be used to develop context-specific solutions for implementing iCBT [17].

Three directions for future research are proposed. First, studies should aim to corroborate findings from our study in relation to stigma and perceived patient barriers to accessing the service, with clients using iCBT. Second, a larger sample size should be employed across different regions of Eastern Europe to enable both in-country and cross-country analyses to be conducted. Third, in-depth face-to-face qualitative interviews should also be used to develop a more detailed account of organizational readiness at an individual level [22].

### Conclusion

This study aimed to gain a qualitative understanding of organizational readiness for implementing iCBT in CMHCs across Kosovo and Albania. Our study found that almost all participants valued iCBT as a resource for extending the human workforce to meet the needs of stigmatized and geographically isolated people. However, several mental health system inadequacies such as insufficient resource availability (eg, inadequate technological infrastructure, limited human work force) and task management (eg, lack of CBT expertise and supervision) present challenges for the implementation of iCBT. Moreover, longstanding situational factors pertaining to mental health stigma have shaped the way in which CMHCs are accessed. Interviews suggested that the majority of people accessing services were in need of urgent assistance mentally and financially. However, there was very little representation from people with mild to moderate presentations such as depression, who are as a result rendered as both underserved and unserved, potentially compounding the effect on their mental health presentation and severity. This pattern of service use appears to be driven by the pervasive stigma associated with mental illness to which there is no easy solution and which requires extensive resources and time to address. While iCBT offers opportunities to decrease stigma associated with engaging patients to mental health interventions, much work is required to increase the treatment-seeking behaviors of people with depression before iCBT can add value to the CMHC. Organizational readiness for implementing iCBT appears to be low, requiring both mental health care systems and situational factors to be addressed before iCBT can optimally be implemented. We propose that such barriers can be addressed through (1) a public-facing campaign that addresses mental health stigma, (2) service-level adjustments that permit staff with the time, resources, and clinical supervision to deliver iCBT in order to increase organizational readiness and facilitate the effective implementation of iCBT in Albania and Kosovo, and (3) establishing a suitable clinical training curriculum for health care professionals to enable them to provide evidence-based treatments for mental health conditions.

## Abbreviations

CMHC: community mental health center
FGD: focus group discussion
GP: general practitioner
iCBT: internet-based cognitive behavioral therapy
ORIC: organizational readiness for implementing change
